# Supplementary material for: Integration of pre-trained protein language models into geometric deep learning networks
Source: Commun Biol. 2023 Aug 25;6:876. doi: 10.1038/s42003-023-05133-1 (PMC10457366; doi:10.1038/s42003-023-05133-1)
Supplement: Supplementary file 1 — Supplemental Information [file 42003_2023_5133_MOESM1_ESM.pdf]

Supplementary Information

Fang Wu, Dragomir Radev and Jinbo Xu

Supplementary Note 1

Here we provide the description of all datasets used in the main text.

Sequence Position Identification

We use a subset of 3243 high-resolution structures from the PDB and adopt a random split of 2643/330/330 for train/val/test. The distribution of the number of residues is plotted in Supplementary Figure 1. For the train set, the maximum and the minimum number of residues are 6248 and 43 separately. The mean and standard deviation of the number of residues are 389.9 and 385.7. For the validation set, the maximum and the minimum number of residues are 9999 and 59 separately. The mean and standard deviation of the number of residues are 454.3 and 772.0. For the test set, the maximum and the minimum number of residues are 2326 and 59 separately. The mean and standard deviation of the number of residues are 383.2 and 286.2.

Model Quality Assessment

The Critical Assessment of Structure Prediction (CASP) [1] is a long-running international competition held biennially, of which CASP13 is the most recent that addresses the protein structure prediction problem by withholding newly solved experimental structures. Mirroring the setup of the competition, we follow [2] and split the decoy sets based on target and released year. We choose CASP11 as the test set, as the targets in CASP12-13 are not fully released yet. This leads to a dataset split of 25400/2800/16014 for train/val/test.

Protein-protein Rigid-body Docking.

We use the DB5.5 database, which is obtained from https://zlab.umassmed.edu/benchmark/. It is randomly partitioned in train/val/test splits of sizes 203/25/25. It is worth mentioning that DB5.5 also includes unbound protein structures, however, which mostly show rigid structures.

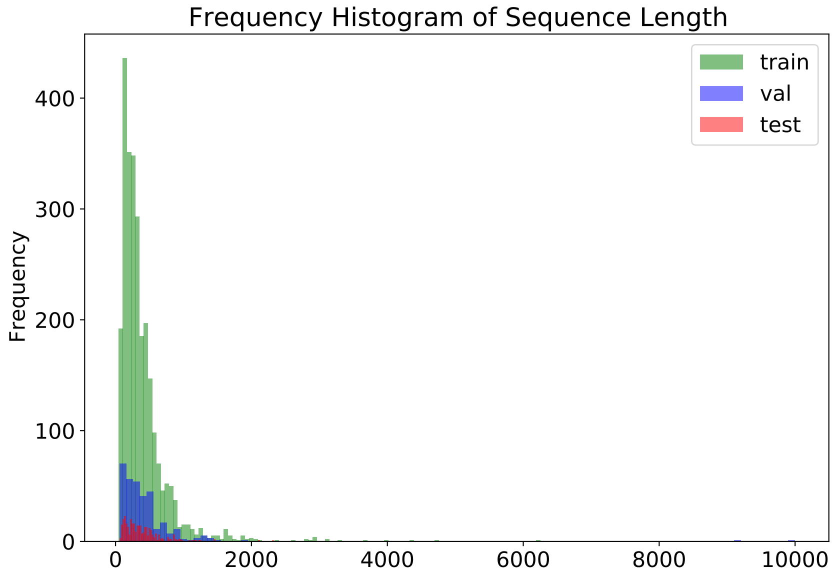

Supplementary Figure 1: The histogram of the sequence length

Protein-protein Interface Prediction

We adopt a part of the DIPS database and use a data split of 12216/1526/1526 for train/val/test. Each complex is an ensemble, with the bound ligand and bound receptor structures forming 2 distinct sub-units of the ensemble. We then define the neighboring amino acids as those with any α-carbon within 8Å of one another. These neighbors are then included as the positive samples, with all other residues as negatives. At prediction time, we attempt to re-predict which possible residues are positive or negative. In other words, we desire to determine whether each residue is located in the binding pocket. AUROC of those predictions is used as the metric to evaluate performance.

Ligand Affinity Prediction

PDBbind contains X-ray structures of proteins bound to small molecules and peptide ligands, We use the dataset mined from PBDbind by [2], which has two splits based on 30% and 60% sequence identity thresholds, respectively. Splitting using 30% sequence identity results in train/val/test of 3507/466/490, while splitting using 60% sequence identity results in train/val/test of 3678/460/460.

Supplementary Note 2

We elucidate the details of backbone architecture. For tasks that only require the predictive model to output a scalar for the protein/complex or each residue including model quality assessment, protein-protein interface prediction, and ligand binding affinity prediction, we select GVP-GNN, EGNN, and Molformer as the backbone architecture. For more complicated tasks that require more complex computational processes such as protein-protein rigid-body docking, we use specific models to address them like Equidock.

GVP-GNN.

GVP-GNN [3, 4] is an equivariant GNN in which all node and edge embeddings are tuples $(\mathbf{s}, \mathbf{V})$ of scalar feature and geometric vector features. Message and update functions are parameterized by *geometric vector perceptrons* (GVPs) – modules mapping between tuples $(\mathbf{s}, \mathbf{V})$ while preserving rotation equivariance. Its computational process is described in Algorithm 1, where $\mathbf{s}$ and $\mathbf{V}$ correspond to the node embedding $\mathbf{h}$ and coordinates $\mathbf{x}$ separately.

Algorithm 1 GVP-GNN

Input: Scalar and vector features $(\mathbf{s}, \mathbf{V}) \in \mathbb{R}^n \times \mathbb{R}^{\nu \times 3}$.
Output: Scalar and vector features $(\mathbf{s}', \mathbf{V}') \in \mathbb{R}^n \times \mathbb{R}^{\mu \times 3}$.
  $h \leftarrow \max(\nu, \mu)$
  $\mathbf{V}_h \leftarrow \mathbf{W}_h \mathbf{V} \quad \in \mathbb{R}^{h \times 3}$
  $\mathrm{V}_\mu \leftarrow \mathbf{W}_\mu \mathbf{V}_h \in \mathbb{R}^{\mu \times 3}$
  $\mathrm{s}_h \leftarrow \|\mathrm{V}_h\|_2$ (row-wise) $\in \mathbb{R}^h$
  $\mathrm{v}_\mu \leftarrow \|\mathrm{V}_\mu\|_2$ (row-wise) $\in \mathbb{R}^\mu$
  $\mathrm{s}_{h+n} \leftarrow \mathrm{concat}\ (\mathrm{s}_h, \mathbf{s}) \in \mathbb{R}^{h+n}$
  $\mathrm{s}_m \leftarrow \mathbf{W}_m\ \mathrm{s}_{h+n} + \mathbf{b} \in \mathbb{R}^m$
  $\mathbf{s}' \leftarrow \sigma\left(\mathrm{s}_m\right) \in \mathbb{R}^m$
  $\mathbf{V}' \leftarrow \sigma^+\left(\mathbf{v}_\mu\right) \odot \mathbf{V}_\mu$ (row-wise multiplication) $\in \mathbb{R}^{\mu \times 3}$
Return: $(\mathbf{s}', \mathbf{V}')$

At its core, GVP-GNN consists of two separate linear transformations $\mathbf{W}_m$ and $\mathbf{W}_h$ for the scalar and vector features, followed by nonlinearities σ, σ^+. An additional linear transformation $\mathbf{W}_\mu$ is inserted before the vector nonlinearity to control the output dimensionality independently of the number of norms extracted. We adopt a 5-layer GVP-GNN with a dropout rate of 0.7 and a ReLU activation function. The number of radial bases in the edge embedding is 16 and the node dimension is set as (100, 16). All implementation codes are downloaded from the official repository in https://github.com/drorlab/gvp.

EGNN.

EGNN [5] achieves equivariance without expensive high-order representations in intermediate layers and also realizes competitive performance. Its Equivariant Graph Convolutional Layer (EGCL) takes the set of node embedding $\mathbf{h}^l = \{\mathbf{h}_i^l\}_{i=1,\ldots,N}$, the coordinate embeddings $\mathbf{x}^l = \{\mathbf{x}_i^l\}_{i=1,\ldots,N}$ and edge information $\mathcal{E} = (e_{ij})$ as input, and then outputs a transformation on $\mathbf{h}^{l+1}$ and $\mathbf{x}^{l+1}$. Concisely, The equations that define this layer are described as follows:

$$\begin{aligned}
\mathbf{m}_{ij} &= \phi_e\left(\mathbf{h}_i^l, \mathbf{h}_j^l, \left\|\mathbf{x}_i^l - \mathbf{x}_j^l\right\|^2, a_{ij}\right), \\
\mathbf{x}_i^{l+1} &= \mathbf{x}_i^l + C\sum_{j\neq i}\left(\mathbf{x}_i^l - \mathbf{x}_j^l\right)\phi_x\left(\mathbf{m}_{ij}\right), \\
\mathbf{m}_i &= \sum_{j\neq i}\mathbf{m}_{ij}, \\
\mathbf{h}_i^{l+1} &= \phi_h\left(\mathbf{h}_i^l, \mathbf{m}_i\right),
\end{aligned} \tag{1}$$

where $\mathbf{h}_i^l \in \mathbb{R}^{nf}$ is the nf_dimensional embedding of node v_i at layer l. a_{ij} are the edge attributes. ϕ_e and ϕ_h are the edge and node operations respectively which are commonly approximated by Multi-layer Perceptrons (MLPs). $\phi_x : \mathbb{R}^{nf} \to \mathbb{R}^1$ is the function that takes the edge embedding $\mathbf{m}_{ij}$ as input from the previous edge operation and outputs a scalar value. C is chosen to be $1/\|\mathcal{N}_i\|$, which divides the sum by its number of neighboring (connected) elements. We choose a 4-layer EGNN with a Siwsh activation function as a non-linearity. The number of the dimension for edges is 16 and residue connections are used. All implementation codes are downloaded from the official repository in https://github.com/vgsatorras/egnn.

Molformer.

Molformer [6] is a variant of Transformer that employs a heterogeneous self-attention layer to differentiate the interactions between multi-level nodes. Here we use a weaker version of Molformer. To be explicit, we do not extract any sort of motifs from either protein or small molecules. Besides, we abandon the multi-scale self-attention mechanism and the Attentive Farthest Point Sampling (AFPS) for more efficient computations and only use the global features. Even though we pick up a simplified form of Molformer, its performance is competitive with or even outperforms EGNN and GVP-GNN on all tasks. The Molformer architecture has 2 layers, 4 heads, a hidden-layer dimension of 1280, and a dropout rate of 0.1. All implementation codes are downloaded from the official repository in https://github.com/smiles724/molformer.

EquiDock.

Equidock [7] predicts the rotation and translation to place on of the proteins at the right docked position relative to the second protein. It adopts an Independent E(3)-Equivariant Graph Matching Network (IEGMN), which extends

both Graph Matching Networks (GMN) and EGNN. It performs node coordinate and feature embedding updates for an input pair of protein graphs and uses inter- and inter-node messages, as well as E(3)-equivariant coordinate updates. The backbone IEGMN has 5 layers and no dropout. It uses LeakyReLU as the activation function. and does not use distance as an edge feature. All implementation codes are downloaded from the official repository in https://github.com/octavian-ganea/equidock_public.

Supplementary Note 3

We run all experiments on 2 A100 GPUs, each with a memory of 80G. For MQA, PPRD and PPI, we use residue-level graphs for protein representation learning. For PPRD, models are trained using Adam with a learning rate of 2e-4 and early stopping with a patience of 30 epochs. For MQA, PPI, and LBA, models are trained using Adam with a learning rate of 1e-4 and early stopping with a patience of 8 epochs. A Plateau learning rate scheduler is applied with a factor of 0.6, patience of 5, and a minimum learning rate of 5e-7. The batch size is 32 if no out-of-memory (OOM) error is not triggered, otherwise, we adopt a batch size of 16. The maximum epoch is set as 200.

For PPRD, we randomly assign the roles of ligands and receptors during training. For PPI, as mentioned before, we formulate interface as the residues whose least distances to their counterpart protein are within 8 Å. For LBA, we only use the residues within a distance of 6 Å from the ligand (*i.e.*, the pocket) following [2]. We build heterogeneous molecular graphs where nodes for proteins are residue-level and nodes for ligands are atom-level. Here we do not distinguish different atom types and simply regard all atoms as the same group, which is denoted as the new 'LIG' pseudo residue class.

For the PLMs, we use the ESM-2 with a parameter size of 650M and 33 layers as the default one. It is trained on UR50/D2021_04 and has an embedding dimension of 1280. We extract per-residue representations as the input for each task. For the ablation study, we adopt ESM-2 with parameter sizes of 8M, 35M, 150M, and 3B that are all trained on the same UR50/D2021_04 dataset with different layers of 6, 12, 30, and 36 respectively. For more details, please visit the official website of ESM in https://github.com/facebookresearch/esm.

Supplementary Note 4

Ligand Affinity Prediction

Notably, several prior studies on ligand affinity prediction employ a different data splitting mechanism from [2]. To make a more thorough comparison with those advanced algorithms, we conduct an additional experiment and use PDBbind-v2016, which maintains 4056 protein-ligand instances. Following the setting in [8], the training set contains 3772 complexes while the test set has 285 protein-ligand pairs. As displayed in Supplementary Table 1, EGNN powered by PLMs outperforms all strong baselines.

Method	Pearson Correlations
AGL-Score	0.833
HPC-GBT	0.831
TNet-BP	0.810†
TopBP	0.861†
PerSpect	0.840
OPRC	0.838
DC-GBT	0.843
EGNN + PLM	**0.873**

Supplementary Table 1: Comparison of GGNN to advanced-mathematical-based machine learning methods. Values of baselines are copied from [8] and those marked with † use the PDBbind-v2016 core set, which has 290 samples but is deprecated.

Comparison with Other PLM

Many popular PLMs have been used in bioinformatics, such as ESM [9] and ProtTrans [10]. In the main text, we only evaluate the improvement of ESM-2. Here, we provide an additional experiment to explore the promotion of other types of large-scale PLMs. Specifically, we investigate the effectiveness of the knowledge learned by ProtTrans, which is available at its official GitHub https://github.com/agemagician/ProtTrans. In order to realize a fair comparison, we select ProtBERT with a parameter size of 420M, competitive with the 650M parameter size of ESM-2. As displayed in Supplementary Table 2, no single category of PLM dominates the other. Generally, ESM-2 is more powerful for the ligand binding affinity prediction with a 30% sequence identity split. Meanwhile, ProtTrans is more beneficial for the ligand binding affinity prediction with a 60% sequence identity split. This provides an important insight that researchers are encouraged to try different kinds of PLMs in practice to achieve more robust performance.

Investigation of Multiple Sequence Alignments

The longstanding approach in computational biology has been to make inferences from a family of evolutionary-related sequences by fitting a model to each family independently. This inspires a line of research to consider incorporating multiple sequence alignments (MSA) into the learning paradigm of PLMs. Therefore, in this chapter, we make a comparison with those MSA-based methods and try to explore whether MSA and their co-evolutionary signals provide any significant additional value. Here we leverage the MSA Transformer [11] and use MMseqs2 to fast search corresponding MSAs with a maximum number of 128 for each target protein instance. Due to the limitation of computational resources, we select Swiss-Prot from UniProt as the search base and use GVP-GNN as the backbone. As displayed in Supplementary Table 3, the introduction of MSA significantly improves the performance of GGNN in the

Supplementary Table 2: Comparison of different types of PLMs' effects on the LBA task.

Model	PLM	Ligand Binding Affinity Sequence Identity (30%)			
		RMSD↓	Pearson's Correlation↑	Spearman Correlation↑	Kendall Rank↑
GVP-GNN	No	1.6480 ± 0.014	0.2138 ± 0.013	0.1648 ± 0.009	0.1107 ± 0.012
	ESM-2 (650M)	**1.4556 ± 0.011**	**0.5373 ± 0.010**	**0.5078 ± 0.005**	**0.3495 ± 0.009**
	ProtTrans (420M)	1.5321 ± 0.013	0.4824 ± 0.011	0.4713 ± 0.009	0.3230 ± 0.010
EGNN	No	1.4929 ± 0.012	0.4891 ± 0.017	0.4725 ± 0.008	0.3291 ± 0.014
	ESM-2 (650M)	**1.4033 ± 0.013**	**0.5655 ± 0.016**	**0.5448 ± 0.005**	**0.3790 ± 0.007**
	ProtTrans (420M)	1.4883 ± 0.013	0.5209 ± 0.017	0.5012 ± 0.006	0.3511 ± 0.009
Molformer	No	1.9107 ± 0.018	0.4618 ± 0.014	0.4104 ± 0.011	0.2812 ± 0.019
	ESM-2 (650M)	1.6028 ± 0.020	**0.5351 ± 0.017**	**0.5372 ± 0.015**	**0.3758 ± 0.016**
	ProtTrans (420M)	**1.5300 ± 0.015**	0.5240 ± 0.013	0.5149 ± 0.010	0.3629 ± 0.014
		Sequence Identity (60%)			
GVP-GNN	No	1.5438 ± 0.015	0.6608 ± 0.012	0.6668 ± 0.010	0.4797 ± 0.014
	ESM-2 (650M)	1.5137 ± 0.019	0.6680 ± 0.010	0.6716 ± 0.008	0.4786 ± 0.012
	ProtTrans (420M)	**1.5135 ± 0.014**	**0.6693 ± 0.008**	**0.6745 ± 0.007**	**0.4849 ± 0.011**
EGNN	No	1.5928 ± 0.020	0.6274 ± 0.013	0.6271 ± 0.017	0.4498 ± 0.014
	ESM-2 (650M)	1.5595 ± 0.022	0.6445 ± 0.015	0.6463 ± 0.019	0.4656 ± 0.019
	ProtTrans (420M)	**1.5250 ± 0.020**	**0.6650 ± 0.011**	**0.6643 ± 0.015**	**0.4793 ± 0.016**
Molformer	No	1.8610 ± 0.018	0.5528 ± 0.016	0.5309 ± 0.015	0.3738 ± 0.017
	ESM-2 (650M)	**1.5926 ± 0.024**	**0.6524 ± 0.018**	**0.6528 ± 0.016**	0.4367 ± 0.011
	ProtTrans (420M)	1.6107 ± 0.025	0.6451 ± 0.018	0.6403 ± 0.018	**0.4556 ± 0.013**

Supplementary Table 3: Ablation study of PLMs with and without MSA.

MSA	First Rank Loss↓	Model Quality Assessment Spearman Correlation↑		Pearson's Correlation↑		Kendall Rank↑	
		Mean	Global	Mean	Global	Mean	Global
✗	0.076 ± 0.002	0.4683 ± 0.013	0.7104 ± 0.009	0.5530 ± 0.012	0.6981 ± 0.007	0.3479 ± 0.011	0.5135 ± 0.006
✓	**0.062 ± 0.001**	**0.5379 ± 0.015**	**0.7511 ± 0.011**	**0.6259 ± 0.014**	**0.7207 ± 0.005**	**0.3980 ± 0.009**	**0.5441 ± 0.015**

model quality assessment task. More robust benefits are expected if we use a larger search database.

References

[1] Kryshtafovych, A., Schwede, T., Topf, M., Fidelis, K. & Moult, J. Critical assessment of methods of protein structure prediction (casp)—round xiii. *Proteins: Structure, Function, and Bioinformatics* **87**, 1011–1020 (2019).

[2] Townshend, R. J. *et al.* Atom3d: Tasks on molecules in three dimensions. *arXiv preprint arXiv:2012.04035* (2020).

[3] Jing, B., Eismann, S., Suriana, P., Townshend, R. J. & Dror, R. Learning from protein structure with geometric vector perceptrons. *arXiv preprint arXiv:2009.01411* (2020).

[4] Jing, B., Eismann, S., Soni, P. N. & Dror, R. O. Equivariant graph neural networks for 3d macromolecular structure. *arXiv preprint arXiv:2106.03843* (2021).

[5] Satorras, V. G., Hoogeboom, E. & Welling, M. E (n) equivariant graph

neural networks. In *International conference on machine learning*, 9323–9332 (PMLR, 2021).

[6] Wu, F. *et al.* 3d-transformer: Molecular representation with transformer in 3d space. *arXiv preprint arXiv:2110.01191* (2021).

[7] Ganea, O.-E. *et al.* Independent se (3)-equivariant models for end-to-end rigid protein docking. *arXiv preprint arXiv:2111.07786* (2021).

[8] Liu, X., Feng, H., Wu, J. & Xia, K. Dowker complex based machine learning (dcml) models for protein-ligand binding affinity prediction. *PLoS Computational Biology* **18**, e1009943 (2022).

[9] Rao, R., Meier, J., Sercu, T., Ovchinnikov, S. & Rives, A. Transformer protein language models are unsupervised structure learners. *Biorxiv* (2020).

[10] Elnaggar, A. *et al.* Prottrans: towards cracking the language of life's code through self-supervised deep learning and high performance computing. *arXiv preprint arXiv:2007.06225* (2020).

[11] Rao, R. M. *et al.* Msa transformer. In *International Conference on Machine Learning*, 8844–8856 (PMLR, 2021).
