# Supplementary material for: Integration of pre-trained protein language models into geometric deep learning networks
Source: Commun Biol. 2023 Aug 25;6:876. doi: 10.1038/s42003-023-05133-1 (PMC10457366; doi:10.1038/s42003-023-05133-1)
Supplement: Supplementary file 2 — Description of Additional Supplementary Files [file 42003_2023_5133_MOESM2_ESM.pdf]

Description of Additional Supplementary Files

File name: Supplementary Data
Description: Source data for figures.
