# Supplementary material for: Integration of pre-trained protein language models into geometric deep learning networks
Source: Commun Biol. 2023 Aug 25;6:876. doi: 10.1038/s42003-023-05133-1 (PMC10457366; doi:10.1038/s42003-023-05133-1)
Supplement: Supplementary file 4 — Reporting Summary [file 42003_2023_5133_MOESM4_ESM.pdf]

nature portfolio

Corresponding author(s): Jinbo Xu, Fang Wu

Last updated by author(s): Jun 9, 2023

Reporting Summary

Nature Portfolio wishes to improve the reproducibility of the work that we publish. This form provides structure for consistency and transparency in reporting. For further information on Nature Portfolio policies, see our Editorial Policies and the Editorial Policy Checklist.

Statistics

For all statistical analyses, confirm that the following items are present in the figure legend, table legend, main text, or Methods section.

n/a	Confirmed	
☐	☒	The exact sample size (*n*) for each experimental group/condition, given as a discrete number and unit of measurement
☐	☒	A statement on whether measurements were taken from distinct samples or whether the same sample was measured repeatedly
☐	☒	The statistical test(s) used AND whether they are one- or two-sided<br>*Only common tests should be described solely by name; describe more complex techniques in the Methods section.*
☐	☒	A description of all covariates tested
☒	☐	A description of any assumptions or corrections, such as tests of normality and adjustment for multiple comparisons
☐	☒	A full description of the statistical parameters including central tendency (e.g. means) or other basic estimates (e.g. regression coefficient) AND variation (e.g. standard deviation) or associated estimates of uncertainty (e.g. confidence intervals)
☒	☐	For null hypothesis testing, the test statistic (e.g. *F*, *t*, *r*) with confidence intervals, effect sizes, degrees of freedom and *P* value noted<br>*Give P values as exact values whenever suitable.*
☒	☐	For Bayesian analysis, information on the choice of priors and Markov chain Monte Carlo settings
☒	☐	For hierarchical and complex designs, identification of the appropriate level for tests and full reporting of outcomes
☐	☒	Estimates of effect sizes (e.g. Cohen's *d*, Pearson's *r*), indicating how they were calculated

Our web collection on statistics for biologists contains articles on many of the points above.

Software and code

Policy information about availability of computer code

Data collection	In our study, we use the open-sourced dataset, atom3D, to conduct relevant experiments. It is a unified collection of datasets concerning the three-dimensional structure of biomolecules, including proteins, small molecules, and nucleic acids. It is collected by Ron Lab at Stanford and is public available at this link: https://www.atom3d.ai/.
Data analysis	For model quality assessment, we use the protein structure ranking dataset in atom3D. It relates to predicting the three-dimensional structure of a protein molecule, given its sequence. A total of around 700 protein targets are included, which consist of protein targets from Critical Assessment of Structure Prediction (CASP) 5-13. For protein-protein interface prediction, we use the protein-protein interactions dataset in atom3d, which is curated from DIPS. For ligand bind prediction, we use the ligand binding affinity dataset in atom3d. It is derived from crystal structures and ligand binding data from PDBBind (Wang et al., 2004), a widely-used curated database of protein-ligand complexes with experimental affinities derived from literature. For protein-protein rigid-body docking, we use the same dataset and split as Equidock.

For manuscripts utilizing custom algorithms or software that are central to the research but not yet described in published literature, software must be made available to editors and reviewers. We strongly encourage code deposition in a community repository (e.g. GitHub). See the Nature Portfolio guidelines for submitting code & software for further information.

Data

Policy information about availability of data

All manuscripts must include a data availability statement. This statement should provide the following information, where applicable:

- Accession codes, unique identifiers, or web links for publicly available datasets
- A description of any restrictions on data availability
- For clinical datasets or third party data, please ensure that the statement adheres to our policy

All codes and data are stored and clearly explained at the repository by this link: https://github.com/smiles724/GGNN_Meets_PLM.

Human research participants

Policy information about studies involving human research participants and Sex and Gender in Research.

Reporting on sex and gender	We adopt the widely accepted dataset in atom3d and PDBbind and require not further efforts to collect data by ourselves. As a result, there are no human research participants. We confirm that we do not hire any people to conduct experiments. All of our experiments were implemented and ran on the cloud serve with several A100 GPUS.
Population characteristics	*Describe the covariate-relevant population characteristics of the human research participants (e.g. age, genotypic information, past and current diagnosis and treatment categories). If you filled out the behavioural & social sciences study design questions and have nothing to add here, write "See above."*
Recruitment	*Describe how participants were recruited. Outline any potential self-selection bias or other biases that may be present and how these are likely to impact results.*
Ethics oversight	*Identify the organization(s) that approved the study protocol.*

Note that full information on the approval of the study protocol must also be provided in the manuscript.

Field-specific reporting

Please select the one below that is the best fit for your research. If you are not sure, read the appropriate sections before making your selection.

☒ Life sciences ☐ Behavioural & social sciences ☐ Ecological, evolutionary & environmental sciences

For a reference copy of the document with all sections, see nature.com/documents/nr-reporting-summary-flat.pdf

Life sciences study design

All studies must disclose on these points even when the disclosure is negative.

Sample size	For model quality assessment, we split the decoy sets based on target and released year. We choose CASP11 as the test set, as the targets in CASP12-13 are not fully released yet. This leads to a dataset split of 25400/2800/16014 for train/val/test.<br>For ligand affinity prediction, we have two splits based on 30\% and 60\% sequence identity thresholds, respectively. Splitting using 30\% sequence identity results in train/val/test of 3507/466/490, while splitting using 60\% sequence identity results in train/val/test of 3678/460/460.<br>For protein-protein interface prediction, we adopt a part of the DIPS database and use a data split of 12216/1526/1526 for train/val/test.<br>For protein-protein rigid-body docking, we follow Equidock and the data are randomly partitioned in train/val/test splits of sizes 203/25/25.
Data exclusions	We confirm that no data is excluded in our research.
Replication	We confirm that we have provided very detailed descriptions to reproduce the results of our experiments in the Github repository: https://github.com/smiles724/GGNN_Meets_PLM. We state clearly how to install the necessary computational environment, the procedure to download and process the dataset, the workflow to rerun the code and analysis the results.
Randomization	We adopt the official dataset splits described in atom3d and Equidock.
Blinding	No investigators are recruited in our experiments. So there exists no blinding issue.

Reporting for specific materials, systems and methods

We require information from authors about some types of materials, experimental systems and methods used in many studies. Here, indicate whether each material, system or method listed is relevant to your study. If you are not sure if a list item applies to your research, read the appropriate section before selecting a response.

Materials & experimental systems

n/a	Involved in the study
☒	☐ Antibodies
☒	☐ Eukaryotic cell lines
☒	☐ Palaeontology and archaeology
☒	☐ Animals and other organisms
☒	☐ Clinical data
☒	☐ Dual use research of concern

Methods

n/a	Involved in the study
☒	☐ ChIP-seq
☒	☐ Flow cytometry
☒	☐ MRI-based neuroimaging
